# Efficacy of Monopolar Radiofrequency or Microwave Ablation in Intrahepatic Cholangiocarcinoma: A Retrospective Multicenter Study from Association des Gastro-Entérologues Oncologues (AGEO)

**DOI:** 10.3390/cancers16223838

**Published:** 2024-11-15

**Authors:** Antoine Briot, Germain Bréhier, Anaïs Jaillais, Arthur David, Paul Girot, Noémi Reboux, Alice Boilève, Yann Touchefeu

**Affiliations:** 1 Inserm CIC 1413, Hépato-Gastroentérologie, Institut des Maladies de l’Appareil Digestif (IMAD), CHU Nantes, Nantes Université, 44000 Nantes, France; antoine.briot@chu-nantes.fr; 2 Department of Radiology, CHU Angers, 49100 Angers, France; germain.brehier@chu-angers.fr; 3 Department of Gastroenterology, CHU Tours, 37000 Tours, France; a.jaillais@chu-tours.fr; 4 Department of Radiology, CHU Nantes, 44000 Nantes, France; arthur.david@chu-nantes.fr; 5 Department of Gastroenterology, CHD Vendée, 85000 La Roche sur Yon, France; paul.girot@ght85.fr; 6 Department of Gastroenterology, CHU Brest, 29200 Brest, France; noemi.reboux@chu-brest.fr; 7 INSERM U1279, Oncology Department, Gustave Roussy, 94805 Villejuif, France; alice.boileve@gustaveroussy.fr

**Keywords:** intrahepatic cholangiocarcinoma, survival analysis, radiofrequency ablation, microwave ablation

## Abstract

Background: Several locoregional treatments approaches, including thermoablation, have been tested for the treatment of intrahepatic cholangiocarcinoma (ICC) and have shown encouraging results. However, data are heterogeneous in terms of tumor number, size, and ablation technique. Objective: The aim of this study was to investigate the efficacy and prognostic factors in ICC treated by monopolar radiofrequency (RF) or microwave ablation (MW). Methods: This was a retrospective study including patients treated with RF or MW for ICC in six participating centers. DFS and OS were evaluated by the Kaplan–Meier method and prognostic factors by log-rank test and Cox modeling. Results: From January 2015 to October 2023, 24 patients with 31 nodules were treated with RFA or MW. Overall, 70% had chronic liver disease, with 50% at cirrhosis stage. The median size of lesions was 17 mm (6–35 mm). After a median follow-up of 33 months (5–85), the median DFS was 10.5 months. The median OS was 40.8 months. On univariate and multivariate analysis, only lesion size > 17 mm was associated with a poor OS (HR 3.09; IC [1.02; 9.37] (*p* = 0.04). Conclusions: Monopolar radiofrequency or microwave ablation is an alternative to surgery for small ICCs. Tumors < 17 mm were associated with better OS.

## Simple Summary

Percutaneous destruction may be an option in patients with intrahepatic cholangiocarcinoma ineligible for surgery. However, published data are heterogeneous with regard to the number and size of tumors or the technique used (e.g., unipolar or multipolar thermoablation). The aim of this retrospective multicenter study was to evaluate the results of percutaneous destruction of cholangiocarcinoma in a homogeneous population (one to two lesions, maximum tumor size 35 mm) treated with monopolar radiofrequency or microwave alone. In this series of 24 patients, after a median follow-up of 33 months (5–85), median DFS was 10.5 months and median OS was 40.8 months. On univariate and multivariate analysis, only lesion size > 17 mm was associated with poor OS (HR 3.09; IC [1.02; 9.37] (*p* = 0.04). In conclusion, monopolar radiofrequency or microwave ablation is an alternative to surgery for small ICCs.

## 1. Introduction

Biliary cancers are a group of malignant tumors that arise from the biliary epithelium. They account for 3% of all gastrointestinal cancers, with an estimated incidence of fewer than six cases per 100,000 population [1]. Depending on their anatomical location in the biliary tree, they are classified as intrahepatic, perihepatic, and distal cholangiocarcinoma and gallbladder adenocarcinoma.

Intrahepatic cholangiocarcinoma (ICC) accounts for 15% of primary liver cancers. Its incidence is increasing rapidly in Western countries [2]. The causes of intrahepatic cholangiocarcinoma are diverse and vary by geographical area, but share a common mechanism of chronic biliary or hepatic inflammation. Chronic liver diseases (viral, alcoholic, or related to metabolic syndrome) are now well-established risk factors [3]. Improved morphological monitoring of chronic liver disease and recommendations for liver biopsy in cases of suspected hepatocellular carcinoma should lead to more frequent identification of ICC [4].

For localized tumors, surgical resection is the standard of care. However, only 20–30% of these patients are candidates for surgery due to general health, portal hypertension, or comorbidities [5]. If the tumor is resected, adjuvant therapy with capecitabine is recommended [6]. For unresectable disease, systemic therapy with a combination of gemcitabine, cisplatin, and durvalumab is the standard of care [7,8]. However, in the case of disease confined to the liver, there is scope for locoregional treatments such as thermal ablation by radiofrequency (RF) or microwave (MW) ablation, but data in this setting remain limited and heterogeneous.

The main objective of this study was to evaluate the efficacy of thermal ablation in ICC and to investigate potential prognostic factors for disease-free survival (DFS) and overall survival (OS).

## 2. Patients and Methods

### 2.1. Patients

All patients with histologically documented intrahepatic cholangiocarcinoma treated by thermal ablation (radiofrequency or microwave ablation) from January 2015 to October 2023 at six participating centers were retrospectively included. All cases were discussed in multidisciplinary meetings, and patients were considered not eligible for surgical resection. Clinical, biological, and imaging characteristics were collected using electronic medical records. The data were last updated in October 2023.

### 2.2. Treatment

Monopolar radiofrequency ablations were performed using an intratumor placement of internally cooled probes or needles with expandable curved electrodes. Microwave ablations were performed using various ablation systems, with duration and power de-pending on tumor size. If necessary, a hydrodissection with glucose or physiological serum or a carbodissection with carbon dioxide was carried out in order to protect neighboring structures. Track ablation was systematically performed during needle withdrawal for both techniques. Correct ablation of the target lesion was confirmed by a contrast-enhanced CT acquisition performed immediately after ablation. In some patients, the procedure was conducted using a laparoscopic or laparotomic approach.

### 2.3. Outcome Assessment

DFS was defined from the day of the treatment until disease progression or death, whichever occurred first. Patients who were alive and did not experience any of these events were censored at the date of the last follow-up. Overall survival (OS) was defined from the day of the treatment until death (from all causes).

### 2.4. Statistics

DFS and OS were calculated according to the Kaplan–Meier method. Prognostic factors of OS and DFS were analyzed using univariate analysis with the log-rank non-parametric method. Variables with a *p*-value < 0.05 or clinically relevant with *p* < 0.2 on univariate analysis were included in a multivariate Cox regression. Data were checked for multicollinearity with the Belsley–Kuh–Welsch technique and proportional hazards were checked according to Schoenfeld residuals. The alpha risk was set to 5%. Statistical analysis was performed with EasyMedStat (version 3.28; www.easymedstat.com).

## 3. Results

### 3.1. Study Population

Twenty-four patients with intrahepatic cholangiocarcinoma treated with thermal ablation were included. Baseline patient and tumor characteristics are summarized in Table 1. The majority of patients were male (75%), with a median age of 67.2 years (54–85), and all had good overall health (ECOG 0–1).

Overall, 70% of patients had chronic liver disease, including 54% with cirrhosis. All patients with cirrhosis had a Child–Pugh A score.

Three patients had a history of treated histologically proven HCC. Five had previously been treated for ICC (resection *n* = 3, selective intra-arterial radiotherapy *n* = 2).

Patients had one (*n* = 14, 58%) or two (*n* = 10, 42%) ICC lesions. The median size was 17 mm (6–35 mm).

### 3.2. Treatment

Twenty-two procedures were performed percutaneously and two by laparoscopy. The treatment used was RF in 58% of cases and MW in 42% of cases. Treatment was ultrasound guided in 54% of cases (*n* = 13) and CT-guided in 46% of cases (*n* = 11). Only two procedures required hydrodissection protection due to the proximity of another organ. Early follow-up CT was performed within 24 h of the procedure in 67% of cases (*n* = 16). Five patients (21%) received adjuvant chemotherapy (capecitabine = 2, gemcitabine *n* = 2, and FOLFOX regimen *n* = 1).

### 3.3. Safety

The only reported complication was a grade 2 pneumothorax (CTCAE V6.0), which resolved favorably after percutaneous drainage.

### 3.4. Disease-Free Survival

**Figure 1:**
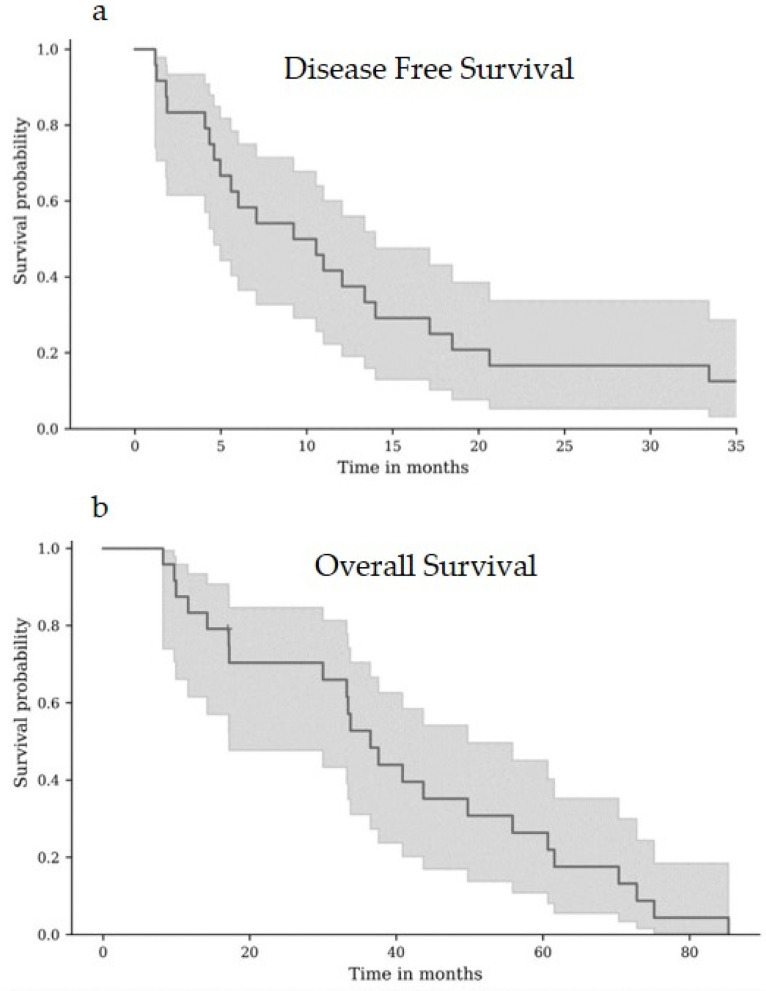
Kaplan–Meier curves for disease-free survival (**a**) and overall survival (**b**).

Median follow-up was 33 months (5–85). Median DFS was 9.2 months (Figure 1a). DFS was 58.3% (95% confidence interval (CI): 36.4–75) at 6 months and 37.5% (95% CI: 19.0–56.0) at 12 months. During follow-up, recurrence occurred in 21 patients (87%). The recurrence was local (less than 1 cm from the thermal ablation zone) in eight patients (38%), distant in ten patients (47%), and both in three patients (14%).

### 3.5. Overall Survival

The median OS was 36.5 months (Figure 1b). OS was 91.7% (95% CI: 70.6–97.8) at 9 months and 79% (95% CI: 57–90.8) at 12 months. At the end of follow-up, 17 patients (74%) had died. Death was related to the disease in 15 cases (88%). In the three patients with surgically treated recurrence, DFS was 6.0, 10.8, and 18.5 months.

### 3.6. Prognostic Factors for DFS

DFS at 6 months for lesion size ≤ 20 mm was 66.7% (95% CI: 40.4–83.4) and 50.0% (95% CI: 11.1–80.4) at 12 months. DFS at 12 months for lesion size ≤ 20 mm was 44.4% (95% CI: 21.6–65.1) and 33.3% (95% CI: 4.6–67.6) at 12 months. The cut-off of 20 mm did not enable statistical comparisons because of an imbalance in the number of patients in each group (16 patients in the ≤20 mm group and 8 in the >20 mm group). For the univariate and multivariate analyses, we used a median tumor size 17 mm as a cut-off.

On univariate and multivariate analysis, no statistically significant prognostic factors were identified. There was a trend towards superiority of MW over radiofrequency (HR 0.419; IC 95% [0.14; 1.25], *p* = 0.12) (Table 2).

### 3.7. Prognostic Factors for OS

OS at 24 months for lesion size ≤ 20 mm was 83.3% (95% CI: 56.8–94.3) and 50.0% (95% CI: 11.1–80.4) at 12 months. OS at 60 months for lesion size ≤ 20 mm was 40.2% (95% CI: 17.3–62.2) and 16.7% (95% CI: 0.8–51.7) at 12 months.

On univariate and multivariate analysis, only the lesion size was associated with a poor OS for lesions > 17 mm (HR 3.09; IC [1.02; 9.37]) with a significant difference (*p* = 0.04) (Table 3).

### 3.8. Treatments After Recurrence

Of the 21 patients with recurrence, 19 received subsequent treatment (thermal ablation *n* = 9, chemotherapy *n* = 6, selective intra-arterial radiation therapy *n* = 2, external beam radiotherapy *n* = 2).

## 4. Discussion

In this study, after a median follow-up of 33 months, patients with ICC treated with thermal ablation had median DFS and OS of 9.2 months and 36.5 months, respectively. Recurrence occurred in 21 patients (87%). These results are consistent with two meta-analyses. In a meta-analysis including 645 patients treated with thermal ablation, the pooled median OS was 30.2 months (95% CI 21.8–38.6). In this meta-analysis, only 31.1% had underlying cirrhosis and 51% had previously undergone surgery [9]. In another meta-analysis including 917 patients treated with WM or RF, pooled OS rates were 82.4% at 1 year, 42.1% at 3 years, and 28.5% at 5 years [10].

In our study, tumor size was prognostic for OS, with a cut-off of 17 mm. Compared to other studies, the size of the lesions was homogeneous, with no lesion larger than 35 mm in diameter. In another study of 27 patients with cirrhosis, the median size was 21 mm (11–45 mm), and median DFS and OS were 10.1 and 30.6 months, respectively [11]. Another study included 29 patients with a median lesion size of 17 mm (5–48 mm), and tumor size < 2 cm was associated with better PFS [12].

For localized and resectable forms, the standard of care is surgical resection, but only 20% to 30% of patients are eligible for resection at the time of diagnosis [5]. Despite surgical advances, it remains a major surgery with significant morbidity and mortality and a high incidence of postoperative liver failure [13]. Several series evaluating surgical resection for ICC have reported median survival ranging from 9 to 40 months [13,14]. Lesions in surgical series are larger, with less frequently associated cirrhosis. Tumor size has an impact on prognosis, with 1-year and 5-year OS rates of 93% and 53% for lesions < 5 cm, and 64% and 28% for lesions > 5 cm [14].

Recurrence was distant in 10 patients and both local and distant in 3 patients, giving a total of 54% of patients. Similar results were found in a retrospective cohort of 71 patients, with a distant recurrence rate of 59%. In this study, local recurrence was lower in multibipolar versus monopolar RF [15]. However, multipolar radiofrequency is currently only available in a few expert centers. In patients with resected ICC, the recurrence rate remains between 50% and 70% of patients, with a 5-year OS rate of approximately 20–35% [16]. In our study, only three patients had previously been treated by surgical resection. DFS were 6.0, 10.8 and 18.5 months respectively. In another series of 20 patients with recurrent ICC treated with RF after curative resection, the median OS was 27.4 months [17]. One study evaluated the role of repeat resection versus thermal ablation in 77 patients with recurrent ICC. There were no differences (*p* = 0.99) in OS or DFS between resection and thermal ablation, except in the group with tumors > 3 cm, where OS was better in the resection group. Morbidity was higher in the resection group [18]. In a series of 71 patients, 36 were treated with RF and 35 with MW, and DFS and OS were superior in patients treated with MW. Tumor size and MW technique were the two independent prognostic factors on multivariate analysis [19]. In our study, there was a trend towards better DFS with MW ablation on multivariate analysis, but the number of patients in our study was lower.

Our study had the advantage of including a homogeneous population (underlying liver disease, presence of one or two nodules), lesions (no large tumors), and treatment (monopolar treatment only). This study has several limitations. It is a retrospective study with a small number of patients. It can be hypothesized that the increase in the number of biopsies for the diagnosis of hepatocellular carcinoma will allow some misdiagnosis of tumors treated by thermal ablation to be avoided. In this retrospective study, some data could not be analyzed, such as potential biological biomarkers, and toxicity may have been underestimated. The evaluation of thermal ablation for ICC in a prospective and randomized trial seems difficult to imagine. In our study, only five patients received adjuvant chemotherapy. The impact of chemotherapy in this setting remains to be determined, but it is also unlikely that a randomized trial could be set up.

## 5. Conclusions

In conclusion, monopolar radiofrequency or microwave ablation is safe for the treatment of ICC and is an option for patients with small tumors and ineligible for surgery. These patients should be discussed in multidisciplinary meetings with competence in interventional radiology.

## Figures and Tables

**Table 1:** Patients’ characteristics.

Age (Years (Median, Range))	67.2 (54–85)
Sex	
- Male, *n* (%)	18 (75)
- Female, *n* (%)	6 (25)
Baseline performance status	
- 0, *n* (%)	16 (67)
- 1, *n* (%)	8 (33)
Obesity (BMI > 30), *n* (%)	8 (33)
Diabetes, *n* (%)	17 (71)
Fibrosis	
- No, *n* (%)	7 (29)
- Fibrosis (F1–F3), *n* (%)	4 (17)
- Cirrhosis, *n* (%)	13 (54)
Child–Pugh score (if cirrhosis)	
- A5, *n* (%)	6 (46)
- A6, *n* (%)	7 (54)
History of HCC histologically proven and treated, *n* (%)	3 (12)
Tumors markers (median, range)	
- aFP	14 (2–56)
- Ca 19.9	179 (1–1308)
- ACE	7 (1–23)
TP (median, range)	84 (30–109)
Platelets counts at diagnosis (median, range) (G/L)	177 (52–403)
Albumin (median, range) (g/L)	39 (31–47)
Bilirubin (median, range) (μmol/L)	13 (5–62)
Number of lesions	
1. *n* (%)	14 (58)
2. *n* (%)	10 (42)
Size of lesion (median, range) (mm)	17 (6–35)
Type of treatment	
- RF, *n* (%)	14 (58)
- MW, *n* (%)	10 (42)
Type of guidance	
- US	13 (54)
- CT	11 (46)
Adjuvant chemotherapy (*n* = 5)	
- Capecitabine	2
- Gemcitabine	2
- 5FU–oxaliplatin	1
Previous treatment (*n* = 5)	
- Surgery	3
- Selective intra-arterial radiation therapy	2
Neoadjuvant chemotherapy	3
Treatments after recurrence (*n* = 19)	
- Thermal ablation (RF/MW)	9
- Chemotherapy	6
- Selective intra-arterial radiation therapy	2
- External beam radiation therapy	2

**Table 2:** Univariate and multivariate analyses for disease-free survival (DFS).

	Univariate Analysis		Multivariate Analysis		
	Median DFS (Months)	*p*-Value	HR	CI (95%)	*p*-Value
Type of treatment MW RF	11.0 9.2	0.11	0.419	[0.14; 1.25]	0.12
History of HCC Yes No	4.1 10.5	0.73			
Hepatopathy No Fibrosis Cirrhosis	20.6 10.5 9.2	0.855			
Gender Male Female	10.5 5.6	0.921			
Number of lesions 1 2	14 6	0.284			
Lesion size <17 mm >17 mm	9.2 10.5	0.863			
Type of guidance CT US	6 11	0.491			
Previous surgical treatment No Yes	11 10.5	0.918			
Adjuvant chemotherapy No Yes	9.2 18.5	0.456			

**Table 3:** Univariate and multivariate analyses for overall survival (OS).

	Univariate Analysis			Multivariate Analysis	
	Median OS (Months)	*p*-Value	HR	CI (95%)	*p*-Value
Type of treatment MW RF	40.8 43.7	0.64			
History of HCC Yes No	55.9 37.6	0.475			
Hepatopathy No Fibrosis Cirrhosis	40.8 43.7 37.6	0.543			
Gender Male Female	43.7 17.2	0.35			
Number of lesions 1 2	33.2 55.9	0.529			
Lesion size ≤17 mm >17 mm	55.9 17.2	0.004	3.09	(1.02–9.37)	0.004
Type of guidance CT US	40.8 49.7	0.491			
Adjuvant chemotherapy No Yes	43.7 37.6	0.741			

## Data Availability

The data presented in this study are available in this article.
